# Nursing Students’ Emotional State and Perceived Competence During the COVID-19 Pandemic: The Vital Role of Teacher and Peer Support

**DOI:** 10.3389/fpsyg.2021.793304

**Published:** 2022-01-27

**Authors:** Britt Karin Utvær, Hanne Torbergsen, Tove Engan Paulsby, Gørill Haugan

**Affiliations:** ^1^Department of Teacher Education, Norwegian University of Science and Technology, Trondheim, Norway; ^2^Department of Public Health and Nursing, Norwegian University of Science and Technology, Trondheim, Norway; ^3^Faculty of Nursing and Health Sciences, Nord University, Bodø, Norway

**Keywords:** COVID-19 pandemic, digital learning, teacher support, peer support, perceived competence, emotional state

## Abstract

**Background:**

The COVID-19 pandemic has led to the shutdown of society and created sudden and long-lasting changes in teaching practices, forcing many nursing students to study remotely at home. These students’ relatedness with their teachers and peers has been limited and mainly online. Several studies have indicated that students’ emotional states and mental health have been negatively affected by the pandemic, representing a serious challenge for many countries. Because they use only digital tools, online students have perceived a decline in teacher and peer support. Likewise, these students have reported feelings of sadness, loneliness, anxiety, and stress, affecting their learning and competence development.

**Aims:**

To investigate the associations between peer support, teacher support, emotional state, and perceived competence in nursing students during the pandemic.

**Methods:**

This cross-sectional study collected quantitative survey data from 329 nursing students at a large university in Norway. Structural equation modeling (SEM) was used to test seven associations among peer support, teacher support, emotional state, and perceived competence.

**Results:**

Teacher support had a significant direct effect on perceived competence, while peer support almost had a significant direct effect. However, the emotional state was directly affected by peer support and had a direct impact on perceived competence. Hence, teacher and peer support is important to nursing students’ perceived competence.

**Conclusion:**

During the COVID-19 pandemic, both peer and teacher support can significantly support students’ competence development. Therefore, students should utilize the support of their teachers and peers in a structured manner to bolster their competence development.

## Introduction

Globally, the COVID-19 pandemic has led to the shutdown of societies and created sudden and long-lasting changes in universities’ teaching practices: universities have closed their campuses, and much teaching has been online. Regardless of the subject and country, all university students have been impacted. Research indicates that students’ emotional states and mental health have been negatively affected by the pandemic situation. Crucially, rates of depression, anxiety, sadness, and loneliness have increased among young university students (e.g., Islam et al., 2020; Kaparounaki et al., 2020; Loades et al., 2020; Commodari et al., 2021; Idoiaga Mondragon et al., 2021; Kakuchi, 2021; Sivertsen, 2021; Suresh et al., 2021). The proportion of Norwegian university students struggling with serious mental illness has increased from 32% in 2018 to 45% in 2021. Likewise, the proportion of students longing for company with others (e.g., feeling excluded or isolated) has increased from 30% in 2018 to 54% in 2021 (Sivertsen, 2021). Studies on nursing students have reported similar results (Labrague et al., 2020; Gao et al., 2021; Miao et al., 2021; Patelarou et al., 2021).

Moreover, recent studies indicate that nursing students’ perceived stress and anxiety have increased in response to the pandemic; for example, many nursing students are worried about the risk of infection (Aslan and Pekince, 2020; Savitsky et al., 2020; Fitzgerald and Konrad, 2021). Registered nurses (RNs) and nursing students have experienced severe stress caused by increased work schedules (Gómez-Ibáñez et al., 2020; Shaukat et al., 2020). Conversely, during their nursing education, many students have part-time jobs at the hospital, nursing home, etc., alongside their studies, which contributes to necessary income. However, due to COVID-19 restrictions, many nursing students have had to stop their part-time work, which subsequently has led to economic uncertainty (Gómez-Ibáñez et al., 2020; Swift et al., 2020). In addition, nursing students’ stress and anxiety levels have been influenced by worries about completing their programs on time, handling new software and applications, dealing with academic workloads (e.g., new assignments), and receiving online assessments in response to lockdowns and new restrictions (Gallego-Gómez et al., 2020; Swift et al., 2020; Amerson et al., 2021; Fitzgerald and Konrad, 2021).

Although the pandemic has impacted all university students, this study focused solely on nursing students. Nursing students are particularly vulnerable to the pandemic, signifying the need for support. First, studies indicate that clinical training is a highly stressful component in nursing curricula (e.g., Shaban et al., 2012; Alzayyat and Al-Gamal, 2014). In contrast to other university studies, 50% of nursing education comprises clinical studies; that is, nursing students work at different hospital wards and in the municipality health care, including nursing homes, etc., requiring close contact with vulnerable individuals. During the pandemic, this is still the case. Accordingly, these students are more prone than the general student body to being infected by the virus, spreading it, and unwillingly being a reason for another person’s illness and potential death. It is rational that young, inexperienced students find this situation stressful and concerning. Second, nursing students’ educational situations during the pandemic have been extraordinary; while accomplishing their educational demands, they have also been prone to being infected, accompanied by the anxiety of spreading the virus to their patients, family, and friends. In this demanding situation, their clinical competence has been assessed and graded as pass or fail by their clinical study. Moreover, since the campuses worldwide have periodically been closed, nursing students’ have had limited access to simulation centers to practice their clinical skills. Plausibly, this embodies a sense of intensified pressure and tension. Third, previous research highlights that nursing students generally report high stress levels (Alzayyat and Al-Gamal, 2016; Labrague et al., 2017) and higher stress levels than other health students (Stecker, 2004), as well as the general student population (Barlett et al., 2016).

During the COVID-19 pandemic, nursing students’ relatedness with their teachers and peers has been limited and mainly digital; teachers worldwide have made extensive use of digital teaching tools (Daniela and Visvizi, 2021). Hence, both perceived teacher and peer support have declined. Over the decades, the literature has provided strong evidence that support from teachers and peers is essential for students’ motivation, adaptive learning strategies, perceived competence, mental health, and wellness (e.g., Wentzel, 2009, 2017; Federici and Skaalvik, 2014; Ryan and Deci, 2017; Suresh et al., 2021). During the COVID-19 pandemic, the literature identified social support as a protective factor against loneliness among nursing students (Labrague et al., 2020). Moreover, nursing students who reported good or excellent teacher support showed fewer symptoms of anxiety and stress and were less concerned about being on track to graduate compared to students reporting poor teacher support. In addition, students with peer support were likelier to approve of their teacher’s response and approach to their education (Fitzgerald and Konrad, 2021). In this context, this study evaluates how perceived teacher and peer support relate to nursing students’ emotional states and perceived competence.

Typically, studies on support in educational settings target emotional and instrumental support (Semmer et al., 2008; Wentzel, 2009; Federici and Skaalvik, 2014). Emotional support is characterized by empathy, friendliness, respect, encouragement, and caring, whereas instrumental support is typified by tangible support. For instance, when teachers provide guidance, they can also provide instrumental support by helping their students understand the content or manage technical issues (Federici and Skaalvik, 2014; Skaalvik and Skaalvik, 2015). Students’ perceptions of emotional support relate to their feelings of belonging, relatedness, and connectedness (e.g., Ryan and Deci, 2017). For instance, Fedesco et al. (2019) measured students’ sense of belonging and relatedness using items that assessed their sense of being accepted and appreciated by their teachers and peers. In this study, we used two subscales of peer relatedness and teacher relatedness to measure nursing students’ sense of relatedness.

Fedesco et al. (2019) showed that teacher relatedness was the most predictive factor of college students’ interest in the course and self-reported effort. Conversely, peer relatedness did not significantly predict any outcome variables. Similarly, Beachboard et al. (2011) explored how learning communities improve higher education learning outcomes and found that relatedness with peers and faculty was a strong predictor of academic development and job preparation. In addition, Ebert et al. (2019) identified the importance of developing a sense of connectedness in both on- and off-campus learning for nursing and midwifery students. They found that a sense of connectedness with academic staff positively influenced students’ socialization and learning. Moreover, participants expressed a preference to learn from those with whom they had formed connections and relationships and a concern about the increase in online teaching. Nursing students experienced a diminished sense of connectedness due to an overreliance on self-directed learning undertaken in isolation from their peers and teachers.

While relatedness is concerned with how students feel when they interrelate with their teachers and peers, instrumental support typically addresses students’ perceptions of being provided with instrumental resources and adequate practical help (Skaalvik and Skaalvik, 2015). Instrumental aspects are characterized by tangible support. Therefore, nurturing and action-facilitating support may be distinguished from each other (Semmer et al., 2008; Federici and Skaalvik, 2014). To assess peer guidance and teacher guidance, we adapted two subscales of instrumental support developed by Federici and Skaalvik (2014). Research has shown that motivational constructs significantly, directly, or indirectly relate to nurturing students’ relatedness and instrumental aspects, such as help with practical problems and direction on how to perform different tasks. Instrumental support demonstrated the strongest relationship with students’ motivation and predicted lower levels of anxiety (Federici and Skaalvik, 2014). Furthermore, Lee et al. (2021) focused on graduate nursing students and showed that peer support, including subscales of both peer relatedness and guidance, was positively related to emotions of positive achievement and learning satisfaction and negatively associated with negative emotions. In addition, positive emotions were positively related to learning satisfaction, whereas negative emotions were negatively associated with learning satisfaction.

Finally, perceived competence is essential for students to reach their educational goals. Within self-determination theory (SDT), perceived competence, autonomy, and relatedness are assumed to be the three fundamental psychological needs. With respect to an activity or domain, the sense or perception of being competent is important because it facilitates an individual’s goal attainment and provides them with a sense of need satisfaction from engaging in an activity at which they feel effective. Numerous studies have demonstrated that basic psychological needs satisfaction significantly relates to human well-being and wellness (Deci and Ryan, 2000; Ryan and Deci, 2017).

In summary, being a nursing student is usually stressful with or without a global pandemic (Stecker, 2004; Alzayyat and Al-Gamal, 2016; Barlett et al., 2016; Labrague et al., 2017; Amerson et al., 2021; Fitzgerald and Konrad, 2021). On a global scale, however, the pandemic has introduced new types of challenges affecting students’ emotional states, mental health, and learning. Due to the extensive use of digital tools, perceived teacher and peer support has declined. Critically, nursing students have experienced sadness, loneliness, anxiety, and stress, harming their emotional state, well-being, learning, and competence development. At the same time, the healthcare system in many countries, including Norway, has a shortage of registered nurses. The fourth wave of COVID-19 intensified the ongoing crisis and led to burnout for many nurses. As a result, a considerable number of nurses are resigning (Falatah, 2021; NSI Nursing Solutions, 2021). Consequently, in light of the aging population globally, the COVID-19 pandemic, and possibly new pandemics, finding ways to encourage and support young people to complete their nursing education is significant. Therefore, this study focuses on nursing students’ perceived competence in relation to emotional state and perceived teacher and peer support.

### Aims

Hence, by using structural equation modeling (SEM), this study aims to provide novel knowledge about nursing students’ perceived competence, their emotional state, and their perceived teacher and peer support. The research questions are as follows:

1. Does perceived teacher and peer support directly affect nursing students’ perceived competence?

2. Does perceived teacher and peer support indirectly affect nursing students’ perceived competence due to the mediation of their emotional state?

**FIGURE 1:**
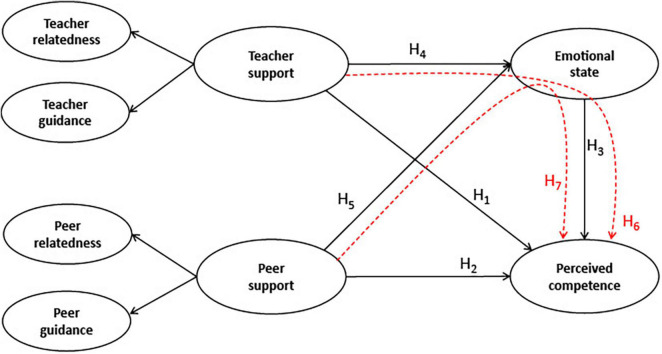
Representation of the hypotheses (H_1_-H_7_); direct relationships represented in black, indirect in red.

Based on the theoretical and empirical knowledge of the associations between perceived competence, emotional state, and perceived teacher and peer support, we hypothesized that support by a teacher (Wentzel, 2009, 2017; Skaalvik and Skaalvik, 2015; Ryan and Deci, 2017) would directly and indirectly affect nursing students’ perceived competence (H_1_ and H_6_), highlighting the importance of relatedness, practical help, and guidance. Furthermore, in this specific situation, while teaching was digital, the teachers aimed to set students together in different learning teams collaborating on their learning. Accordingly, it is plausible that students supported each other’s learning by caring about each other, establishing friendships, and helping to solve technical problems (Wentzel, 2009, 2017; Skaalvik and Skaalvik, 2015; Ryan and Deci, 2017), all of which affected nursing students’ perceived competence (H_2_ and H_7_). Based on SDT theory (Ryan and Deci, 2017), we also hypothesized a positive direct effect of teacher and peer support on nursing students’ emotional state (H_4_ and H_5_). However, we do not know how this works when teaching becomes primarily digital. Finally, the students’ handling of stress, loneliness, sadness, and anxiety will have better conditions for learning and thereby experience perceived competence (H_3_). Hence, we proposed the following hypotheses, which are portrayed in Figure 1:

1. *Hypothesis 1 (H_1_):* Teacher support directly affects perceived competence.
2. *Hypothesis 2 (H_2_):* Peer support directly affects perceived competence.
3. *Hypothesis 3 (H_3_):* Emotional state directly affects perceived competence.
4. *Hypothesis 4 (H_4_):* Teacher support directly affects students’ emotional states.
5. *Hypothesis 5 (H_5_):* Peer support directly affects students’ emotional states.
6. *Hypothesis 6 (H_6_*): Teacher support indirectly affects perceived competence.
7. *Hypothesis 7 (H_7_):* Peer support indirectly affects perceived competence.

## Materials and Methods

### Participants and Procedure

This study is part of a wider Norwegian study on nursing education that focused on student-active learning methods and students’ study effort, emotional state, motivation, and perceived competence. The wider study included the nursing education program at a large university in Norway, and the university’s management unit gave permission to conduct it. In spring 2020, this cross-sectional study collected survey data on all nursing students, including first-, second-, and third-year students. In autumn 2020, all new first-year students were invited to participate in the survey. The inclusion criteria were thus (1) all nursing students at the actual university and (2) those willing to participate. There were no exclusion criteria.

All nursing students received information about the study via e-mail and announcements on their learning platform (Blackboard), with three reminders to participate. In total, 329 students responded to an online questionnaire. All participation was voluntary, guaranteed to be anonymous, and not compensated. The total sample contained 329 (37%) out of 883 viable students. Missing data were handled listwise, giving an effective sample of 305 students.

### Measures

The scales were included and assessed in the following order: (1) teacher support (including relatedness and guidance subscales), (2) peer support (including peer relatedness and peer guidance), (3) emotional state, and (4) perceived competence.

#### Teacher Support

Teacher support consists of two subconcepts, teacher relatedness and teacher guidance. The subconcept of teacher relatedness was assessed using five items from the Adapted Basic Satisfaction Needs at Work scale (Fedesco et al., 2019). This scale reflects the level of connection that students experience in their interactions with their nursing teachers. Teacher relatedness is characterized by empathy, friendliness, affiliation, and caring, all of which are recognized as important requirements for a sense of belonging, relatedness, and connectedness (Deci and Ryan, 2000; Ryan and Deci, 2017). The items for teacher relatedness included *“I get along with the teachers in the course”* and *“The teachers in the course care about me.”* A previous study reported a Cronbach’s alpha of 0.86 for teacher relatedness (Fedesco et al., 2019).

The teacher guidance subscale featured three items on instrumental support (Wentzel, 2009; Federici and Skaalvik, 2014). Federici and Skaalvik (2014) developed and validated an instrumental support measurement that we adapted to the context of homebound nursing teaching. In the present study, we determined the tangible support that teachers and peers can provide in a digital environment. The items for teacher guidance included *“The teachers help me so that I understand what I should focus on in the actual course”* and *“When technical problems emerge, the teachers help me solve them.”*

#### Peer Support

Two subscales on peer relatedness and peer guidance examined peer support. The peer relatedness subscale contained five items from the Adapted Basic Satisfaction Needs at Work scale (Fedesco et al., 2019). Items for peer relatedness included *“I get along with the peers in the course”* and *“The teachers in the course care about me.”* A previous study reported a Cronbach’s alpha of 0.80 for peer relatedness (Fedesco et al., 2019). The peer guidance subscale featured three items on instrumental support (Federici and Skaalvik, 2014) that were adapted for this study. Items for peer guidance included *“The students help me so that I understand what I should focus on in the actual course”* and *“When technical problems emerge, the students help me solve them.”*

#### Emotional State

This study developed four items to assess students’ emotional states, measuring whether they agreed with different statements concerning their emotional states. The items included “*I have felt more stressed than before”* and *“I have felt sadder than before.”*

#### Perceived Competence

Finally, perceived competence was measured with three items from the Perceived Competence for Learning scale (Center for Self-Determination Theory, 2021). The items are concerned with feelings or perceptions of competence with respect to an activity or domain. In this study, the activity or domain was nursing education during the COVID-19 pandemic. The items included *“I feel confident in my ability to learn the materials in the course”* and *“I am able to achieve my goals in the course.”* The perceived competence for the learning scale has high internal consistency, with an alpha measure of 0.80 (Williams and Deci, 1996). All response categories were accompanied by a seven-point scale that ranged from “Absolutely disagree” (1) to “Absolutely agree” (7) with a midpoint of “Neither” (4). Appendix Table 1 demonstrates the concepts and their indicators, along with some additional values (i.e., mean, standard deviation, skewness, and kurtosis).

### Statistical Analysis

The data were analyzed with descriptive statistics using IBM SPSS version 27 (SPSS, 2021). The respective hypothesized relations between the latent variables of teacher support, peer support, students’ emotional states, and perceived competence were tested with a structural support model (SEM) using Stata 17 (StataCorp, 2021).

Research has indicated that Cronbach’s α cannot be generally trusted as an estimator of reliability (Raykov, 2001). Thus, as shown in Tables 1, 2, composite reliability was estimated using the formula developed by Hair et al. (2010). Thus, a coefficient ≥ 0.7 was sufficient for both reliability coefficients. For the correlation analyses, the *p*-value was set to 1%. Estimates based on SEM analyses commonly include both 5% and 1% *p*-values. Factor loadings<0.32 were poor, ≥ 0.45 fair, ≥ 0.55 good, ≥ 0.63 very good, and > 0.71 excellent (Schermelleh-Engel et al., 2003).

**TABLE 1:** Measurement models for the first- and second-order factor models of teacher and peer support.

Items	Parameter	Satorra-Bentler coef^a^	*t*-value	Bentler-Raykov squared multiple correlation^b^ (*R*^2^)	Composite reliability^d^ (*ρ_c_*)
**Teacher relatedness**					
Teach_rel1	*λ1,1*	0.82	33.44**	0.68	0.89
Teach_rel2	*λ2,1*	0.91	52.42**	0.83	
Teach_rel3	*λ3,1*	0.82	34.98 **	0.68	
**Teacher guidance**					
Teach_guid1	*λ4,2*	0.86	32.02**	0.74	0.83
Teach_guid2	*λ5,2*	0.77	23.35**	0.59	
Teach_guid3	*λ6,2*	0.74	23.25**	0.54	
**Teacher support**					
Teach_rel	γ*1,1*	0.95	20.69**	0.89	0.91
Teach_guid	γ*2,1*	0.89	15.46**	0.78	
**Peer relatedness**					
Peer_rel1	*λ7,3*	0.86	48.06**	0.80	0.92
Peer_rel2	*λ8,3*	0.91	48.01**	0.83	
Peer_rel3	*λ9,3*	0.89	40.77**	0.74	
**Peer guidance**					
Peer_guid1	*λ10,4*	0.74	18.43**	0.55	0.84
Peer_guid2	*λ11,4*	0.90	29.60**	0.80	
Peer_guid3	*λ12,4*	0.75	17.74**	0.57	
**Peer support**					
Peer_rel	γ*3,2*	0.86	20.62**	0.73	0.78
Peer_guid	γ*4,2*	0.75	10.69**	0.56	
T_sup-P_sup^c^	*φ1,2*	0.41	6.24**		

**p-value < 0.01.

*^a^Satorra Bentler completely standardized factor loadings.*

*^b^The Bentler-Raykov squared multiple correlation coefficient: R^2^.*

*^c^The covariance between the second-order teacher support and peer support latent constructs.*

*^d^Composite reliability ρ_c_ = $\frac{{\left(\sum \lambda \right)}^{2}}{{\left(\sum \lambda \right)}^{2}+\sum \left(\theta \right)}$.*

**TABLE 2:** Measurement models for students’ emotional state and perceived competence.

Items	Parameter	Satorra-Bentler coef^a^	*t*-value	Bentler-Raykov squared multiple correlation^b^ (*R*^2^)	Composite reliability^e^ (*ρ_c_*)
**Emotional state**					
Emot1	*λ1,1*	0.58	6.81**	0.33	0.80
Emot2	*λ2,1*	0.63	7.26**	0.40	
Emot3	*λ3,1*	0.62	5.97**	0.39	
Emot4	*λ4,1*	0.78	6.85**	0.61	
**Perceived competence**					
Perc_comp1	*λ5,2*	0.85	28.71**	0.72	0.90
Perc_comp2	*λ6,2*	0.88	35.60**	0.78	
Perc_comp3	*λ7,2*	0.87	35.26**	0.76	
Emot1_Emot2*^c^*	*θδ*	0.65	9.92**		
Emot3_Emot4	*θδ*	0.54	4.07**		
Emotional state-	*φ1,2*	–0.31	−4.05**		
Perceived competence^d^					

**p-value < 0.01.

*^a^Satorra Bentler completely standardized factor loadings.*

*^b^The Bentler-Raykov squared multiple correlation coefficient: R^2^.*

*^c^The covariances between the error terms for Item 1 (stress) with Item 2 (anxiety) and Item 3 (loneliness) with Item 4 (sadness), respectively.*

*^d^The covariance between the emotional state and perceived competence factors.*

*^e^Composite reliability ρ_c_ = $\frac{{\left(\sum \lambda \right)}^{2}}{{\left(\sum \lambda \right)}^{2}+\sum \left(\theta \right)}$.*

#### Model Fit

Since the standard errors were estimated under non-normality, the Satorra-Bentler-scaled chi-squared statistic was applied as a goodness-of-fit statistic. It represents the correct asymptotic mean even under non-normality (Jöreskog et al., 2016). In line with the rule of thumb of the conventional cut-off criteria, the following fit indices were used: the chi-square statistic (χ^2^) such that a small χ^2^ and a non-significant *p*-value correspond to a good fit; χ^2^/degrees of freedom such that a value ≤2 indicates a good fit and ≤3 an acceptable fit; and the root mean square error of approximation (RMSEA) and the standardized root mean square residual (SRMS) such that values<0.05 indicate a good fit, whereas values <0.08 are acceptable. In addition, the comparative fit index (CFI) and the Tucker Lewis index (TLI) were applied, with acceptable fits at 0.95 and 0.90, respectively, and good fits at 0.97 and 0.95 and above (Schermelleh-Engel et al., 2003).

## Results

Among the 305 students, 266 were female (87%), and 39 were male (13%). Moreover, 179 students were in their first year of study (59%), 48 in their second year (16%), and 78 in their third year (26%). Table 3 presents the means (M), standard deviations (SD), Cronbach’s α, and Pearson’s correlation matrix of the latent variables included in the SEM. The correlations between the latent variables ranged from –0.25 to 0.71 in the expected direction. The α-levels for the various measures indicated a good level of inter-item consistency, with Cronbach’s alpha coefficients ranging from 0.82 to 0.91.

**TABLE 3:** The mean, Cronbach’s alpha, and correlation coefficients of the study variables.

Construct	Mean (*SD*)	Items	Cronbach’s alpha	1	2	3	4	5	6
1. Perceived competence	4.50 (1.29)	3	0.90	1					
2. Teacher relatedness	3.98 (1.45)	3	0.89	0.39**	1				
3. Teacher guidance	4.13 (1.37)	3	0.84	0.39**	0.71**	1			
4. Peer relatedness	5.46 (1.31)	3	0.91	0.29**	0.23**	0.25**	1		
5. Peer guidance	5.65 (1.13)	3	0.84	0.21**	0.26**	0.33**	0.56**	1	
6. Emotional state	4.43 (1.45)	4	0.82	−0.25**	−0.15**	−0.14*	−0.21**	–0.13*	1

*p-value < 0.05, **p-value < 0.01. Listwise N = 305, Missing N = 24 (7%).

Moreover, we investigated how teacher and peer support related to nursing students’ emotional states and perceived competence and the inter-relatedness between the two dependent latent variables (emotional states and perceived competence). For this purpose, we estimated two complete measurement models of 12 items (the independent variables) and seven items (the dependent variables). The models were tested with confirmatory factor analysis (CFA) using Stata 17 (StataCorp, 2021).

### The Measurement Models

The first measurement model included 12 items representing teacher relatedness (three items), teacher guidance (items), peer relatedness (three items), and peer guidance (three items) (Table 1). Previous studies have suggested a strong correlation between teachers’ emotional and instrumental support (Federici and Skaalvik, 2014; Morin, 2020). The current study found a strong correlation between teacher relatedness and guidance (*r* = 0.71). Thus, we tested whether teacher support and peer support could be treated as two-dimensional constructs that fit into a second-order factor model.

The second-order measurement model largely revealed a good fit (χ^2^ = 100.12, *p* = 0.000, df = 50, χ^2^/df = 2.06, RMSEA = 0.057, p-close = 0.011, CFI = 0.97, TLI = 0.97, SRMR = 0.147). The standardized factor loadings were significant, ranging from 0.74 to 0.91 for the first-order loadings and from 0.75 to 0.95 for the second-order loadings, all significant at the 1% level (*p* < 0.01). Composite reliability (ρ_c_) was good for all concepts, ranging from 0.78 to 0.92. However, several significant residuals were≥1.96 (63.6%), which emphasized the need for specification. Several significant residuals appeared between the teacher relatedness items and peer relatedness items; the same occurred for the teacher and peer guidance items. Nine of the highest residuals appeared between the three items on teacher guidance and three items on peer guidance, ranging from 3.74 to 5.11. Thus, we included a covariance between the second-order teacher and peer support factors, showing a good fit (χ^2^ = 75.53, *p* = 0.009, df = 49, χ^2^/df = 1.54, RMSEA = 0.042, p-close = 0.331, CFI = 0.98, TLI = 0.98, SRMR = 0.031).

Initially, the measurement model on emotional state (four items) and perceived competence (three items) had a poor fit (χ^2^ = 194.91, *p* = 0.000, df = 13, χ^2^/df = 15.0, RMSEA = 0.213, p-close = 0.000, CFI = 0.85, TLI = 0.75, SRMR = 0.098). The standardized factor loadings were significant, ranging from 0.50 to 0.89 (*p* < 0.01). The composite reliability (ρ_c_) was good for emotional state (*ρ_c_* = 0.80) and perceived competence (*ρ_c_* = 0.90). However, some extremely high modification indices indicated misspecification. Hence, a covariance between the “Emotional state1 (stress)” and “Emotional state2 (anxiety)” items (MI = 159.0) as well as the “Emotional state3 (loneliness)” and “Emotional state4 (sadness)” items (MI = 154.3) would considerably improve the measurement model. The inter-item correlations between these pairs of items were 0.77 and 0.76, respectively. Thus, we allowed two correlated error covariances, resulting in values of 0.65 and 0.54, respectively, to considerably improve the fit (χ^2^ = 13.55, *p* = 0.259, df = 11, χ^2^/df = 1.15, RMSEA = 0.027, p-close = 0.723, CFI = 0.99, TLI = 0.99, SRMR = 0.018). Table 2 contains the factor loadings, *t*-values, *R*^2^-values, and *ρ_c_*-values of the measurement model, including covariances for two correlated error terms.

### The Structural Model

**FIGURE 2:**
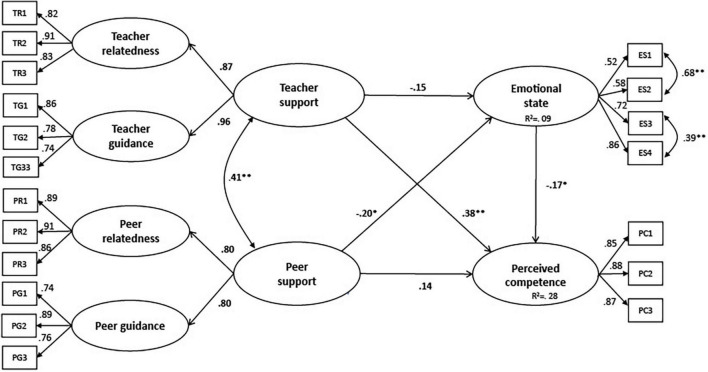
The estimated SEM model. Fit indices: χ^2^ = 161.49, *p* = 0.103, df = 140, χ^2^/df = 1.2, RMSEA = 0.022, p-close = 0.996, CFI = 0.99, TLI = 0.99, SRMR = 0.038. *p-value < 0.05, **p-value < 0.01.

As shown in Figure 2, we used SEM to represent the measurement models with factor loadings, structural regression coefficients, explained variance in the endogenous latent variables, and fit indices.

The SEM yielded a good fit (χ^2^ = 161.49, *p* = 0.103, df = 140, χ^2^/df = 1.2, RMSEA = 0.022, pclose = 0.996, CFI = 0.99, TLI = 0.99, SRMR = 0.038). Table 4 shows the standardized regression coefficients of the direct, indirect (mediated), and total effects between the latent variables.

**TABLE 4:** SEM model: direct and indirect relationships between teacher and peer support, emotional state, and perceived competence.

Constructs	Hypothesis	Direct effects		Hypothesis	Indirect effects		Total effects	
	Parameter	Estimate	*t*-value		Estimate	*t*-value	Estimate	*t*-value
**Perc_competence**								
Teach_support	*^a^*γ2,1 (H_1_)	0.38**	4.91	(H_6_)	0.03	1.39	0.40**	5.31
Peer_support	γ2,2 (H_2_)	0.14	1.80	(H_7_)	0.03	1.12	0.17**	5.83
Emotional state	*^b^*β2,1 (H_3_)	−0.17*	–2.42				−0.17*	–2.42
**Emotional state**								
Teach_support	γ1,1 (H_4_)	–0.15	–1.69				–0.15	–1.69
Peer_support	γ1,2 (H_5_)	−0.20*	–2.05				−0.20*	–2.05

*p-value < 0.05, **p-value < 0.01.

*^a^Gamma (γ): standardized regression coefficients representing direct relationships between the independent (teacher and peer support) and dependent (emotional state and perceived learning) latent constructs.*

*^b^Beta (β): standardized regression coefficients representing direct relationships between the dependent latent constructs.*

As displayed in Figure 2 and Table 4, significant directional paths appeared from teacher support to perceived competence (H_1_: γ2,1 = 0.38^**^) and from emotional state to perceived competence (H_3_: β2,1 = –0.17*). The direct path from peer support to perceived competence was almost significant (H_2_: γ2,2 = 0.14, *t* = 1.80). The same result occurred for the path from teacher support to emotional state (H_4_: γ1,1 = –0.15, *t* = –1.69). However, the direct path from peer support to emotional state was significant (H_5_: γ1,2 = –0.20*), indicating that low peer support was related to higher levels of stress, anxiety, and loneliness.

Looking at the indirect effects, teacher support barely impacted the perceived competence mediated by emotional state (H_6_: 0.03). Thus, the indirect effect was poor and not significant. The total effect of teacher support on perceived competence was strong and appeared directly through relatedness and adequate practical guidance. Finally, peer support related significantly, directly, and indirectly, to the perceived competence that was mediated by the emotional state. The total effect of peer support had a significant positive total effect on perceived competence, even if its direct and indirect effects were not significant (H_7_: 0.03).

## Discussion

Teacher and peer support is important to all university students. During the COVID-19 pandemic, nursing students have experienced increased stress, anxiety, worrying, and several health problems signifying a specific vulnerability, which have necessitated encouragement and support (Aslan and Pekince, 2020; Labrague et al., 2020; Savitsky et al., 2020; Fitzgerald and Konrad, 2021; Patelarou et al., 2021). Simultaneously, to avoid shortages of registered nurses, ensuring that these students complete their education and develop the required competence is crucial. Subsequently, it is fundamental to gain knowledge on how to certify nursing students’ learning and competence development in the midst of a pandemic. Therefore, this study investigated the associations between nursing students’ perceived competence, their emotional state, and perceived teacher and peer support.

Consequently, this study contributes to a holistic perspective that seeks to support the emotional well-being of and competence development in nursing students in three ways: (1) it supplies empirical knowledge to the growing body of emotional and instrumental support literature by exploring nursing students’ experiences of teacher and peer support; (2) it provides empirical insights into the associations between teacher and peer support, emotional state, and perceived competence among nursing students; and (3) it leverages advanced statistical analysis and applies SEM. Based on the results, this study recommends that nursing students should be supported with strategies to promote their emotional well-being and perceived competence during pandemics.

More specifically, this study tested seven hypotheses and found support for three of them (H_1_, H_3_, and H_5_). The results supported the hypothesized relationships between teacher support and students’ perceived competence (H_1_), students’ emotional state and their perceived competence (H_3_), and peer support and students’ emotional state (H_5_). Moreover, the hypothesized relationship between peer support and perceived competence (H_2_) as well as the association between teacher support and emotional state (H_4_) were both close to significant. While assessing the insignificant indirect effects (H_6_ and H_7_), we determined that the total effect of both types of support was important to nursing students’ perceived competence (Table 4). Accordingly, we propose that teacher and peer support should be improved to facilitate nursing competence and the completion of nursing education.

### Experiences of Teacher and Peer Support When Teaching Becomes Digital

Peer support had the highest mean scores, demonstrating its significance for nursing students’ emotional states. When technical problems emerged, instrumental support was especially important and was mostly provided by peers. Hence, the results indicate that the students found support for each other. Amerson et al. (2021) explored mental health among nursing students during a period of virtual learning and showed that students may need to innovate to deal with new assignments that also require new software and applications. Likewise, they noted that the learning time associated with multiple new platforms and software may significantly increase students’ anxiety and stress. We propose that students may find it easier to ask their peers for help with technical problems than their teachers. Moreover, students may direct technological questions to their peers because they perceive them to be more technologically competent than their nursing teachers. A lack of teacher support and knowledge of technological solutions in teaching can trigger students to seek support from each other. Therefore, nursing teachers should facilitate and help students connect, encouraging them to assist each other. This is especially important among first-year students, for whom much information is new; getting to know fellow students, the learning platform, teaching methods, and the clinical work, along with moving to a new city, might be overwhelming. The faculty may involve and organize second- and third-year students in supporting the young students in creating a nurturing and flourishing learning environment. Moreover, the faculty needs to facilitate and support nursing teachers in improving their digital competencies and thus being able to support and guide students as needed.

The students also reported that their peers helped them understand the educational content and directed them to perform different tasks. The pandemic has resulted in the extensive use of digital learning tools, both in theoretical and practical studies. When teaching becomes digital, students may feel insecure about asking their teacher questions, seeking explanations, and requesting guidance. In addition, teachers are digitally present for only a limited time, so students must send a message when they have a request. Moreover, some teachers may digitally observe the practical studies of their students, while their peers may be physically present in the ward. As a result, teacher relatedness can suffer, making peer relatedness and guidance vital. Consequently, teachers must be available for online guidance concerning both theoretical and clinical studies. Likewise, providing students with clear and easily accessible information about how to reach teachers is principal. Furthermore, it is important to introduce the structure and functionality of the learning platform to ensure that students find necessary information, learning material, assignments, etc., feeling self-reliant while using the platform.

Although peer support showed the highest mean score, teacher support was directly and significantly related to perceived competence. Previous research has confirmed that teacher support is essential to students’ motivation, learning, and competence development (Federici and Skaalvik, 2014; Ryan and Deci, 2017). Regarding teacher relatedness, this study determined a mean score in the indecisive range. Evidently, several students felt that they did not have a close relationship with their teacher or that their teacher cared about them. Moreover, the means of teacher guidance were low or indecisive. It is plausible that these students found it difficult to build a vital relationship with the teacher when their education went digital. However, we expected teacher guidance to be stronger, especially for items covering guidance on different work requirements (e.g., written submission, internship assignment, and bachelor’s thesis) and understanding the subject matter.

The results suggest that it was difficult to foster a good learning environment in online classrooms with large student groups. Some students might have found it uncomfortable or even scary to speak loudly in a digital session, while 100–250 others listened. Consequently, these students may not have engaged in questions or reflections, impeding the dialogue. Such issues are reflective of an insecure learning situation that can decrease students’ perceived competence. To prevent students from having unanswered questions and ambiguities at the end of online lectures, teachers can encourage students to extend their stay in the digital room. Teachers can also organize students into smaller groups (e.g., breakout rooms), creating structures for social contact and learning. Furthermore, teachers should encourage students to work together in learning communities and care about each other. Creating reference groups of students and collecting information about students’ experiences concerning the different courses can give valuable feedback. When knowing about challenges or problems, it is easier to provide specific support and implement measures. Even though digital teaching may trigger students to feel unnoticed and inundated; digital teaching has come to stay. Thus, supportive strategies and structures should be developed to enhance students’ empowerment and facilitate their autonomous functionality in the clinic.

In this study, nursing students did not significantly differentiate between instrumental and emotional support. Although there is a clear theoretical and logical distinction between emotional and instrumental support, the literature has similarly demonstrated that students do not clearly distinguish between them. For example, Federici and Skaalvik (2014) and Morin (2020) found correlations of 0.80 and 0.68, respectively, between teachers’ emotional and instrumental support. This study found a correlation of 0.71, which indicates that supportive teachers tended to be supportive in several ways. This finding suggests that the students who received good instrumental support, thus helping them understand the subject matter, perceived their teachers as emotionally supportive as well. The same patterns appeared for peer support. Thus, by providing support, regardless of whether the support is emotional or instrumental, teachers can promote students’ competence development and thereby learning.

Finally, we found a strong correlation between teacher and peer support. When we included covariance between the second-order teacher and peer support latent constructs, this considerably improved the model fit. Students who yearn for support may look for support from both teachers and students. That is, they desire support regardless of who provides it. By supporting student connections, teachers can contribute to a learning environment characterized by support, respect, and care. Ebert et al. (2019) disclosed that nursing and midwifery students preferred learning from those with whom they had formed connections and relationships in both on- and off-campus learning contexts. Aspirations for support might be largely based on relationships rather than on the provider’s role or title.

### Relationships Between Teacher and Peer Support, Perceived Competence, and Emotional State

Concerning perceived competence, about 50% of the nursing students in this study reported that they coped well with the pandemic, whereas about 50% claimed that they did not. Teacher and peer support were vital. In particular, the latter was crucial to the students’ emotional states, which affected their perceived competence and probably their perceived learning as well. The emotional support provided by the teachers affected the students’ perceived competence, mediated by their emotional state.

Research has shown that nursing students report the highest stress levels compared to students in other formalized programs (Stecker, 2004; Barlett et al., 2016). Moreover, nursing students are mostly young women. Both before and during the pandemic, studies have disclosed higher anxiety levels and higher stress levels among female nursing students compared to male nursing students (Aslan and Pekince, 2020; Savitsky et al., 2020). Recently, Gallego-Gómez et al. (2020) revealed a deteriorated self-concept and higher uncertainty among female nursing students. Moreover, young people reported that they experienced more loneliness during the mandatory lockdown, with women having higher odds of loneliness (Labrague et al., 2020; Sivertsen, 2021).

The pandemic has possibly worsened nursing students’ sense of stress, anxiety, loneliness, and sadness. As shown in our study, numerous students (46.8–64.6%) reported that they were more stressed (64.6%), anxious (51.5%), lonely (58.6%), and sad (46.8%) during the pandemic than before, which has negatively influenced their learning and competence development. In this light, peer support seems especially important to students’ emotional states and thereby their learning and perceived competence. This supports the findings of other recent studies among nursing students amid the pandemic (Morin, 2020; Lee et al., 2021).

Prior to the pandemic, nursing students in general reported higher levels of stress and anxiety compared to the overall student body (Barlett et al., 2016; Labrague et al., 2017), inclusive of other health education programs (Stecker, 2004). The nursing program aims to prepare nursing students for professional nursing roles, enhancing their critical thinking and decision-making skills in clinical settings. This clinical component represents about 50% of nursing education and includes demanding learning situations, producing high levels of discomfort, stress, and anxiety (Stecker, 2004; Alzayyat and Al-Gamal, 2016; Labrague et al., 2017). Evidently, the role of a nursing student seems to be quite stressful, representing a special state of vulnerability that calls for further attention and support. The students who coped well with the study situation during the pandemic possibly experienced closer relationships with their peers and teachers than those who struggled. Thus, this support should be recognized as a resource that promotes mental health and learning among nursing students (Labrague et al., 2020). Crucially, students should receive both emotional and instrumental support strategies to bolster their mental health, their competence development, and the completion of their education, which also benefits society (Fitzgerald and Konrad, 2021).

Relatedness, perceived competence, and autonomy—basic human needs as defined by SDT theory—were impaired among nursing students during the pandemic (Deci and Ryan, 2000; Ryan and Deci, 2017). Specifically, peer relatedness was extensively reduced. Combined with reduced teacher–student interaction, this may have caused loneliness, insecurity, anxiety, sadness, and symptoms of depression. These negative emotional states decreased their perceived competence and amplified their fears of not learning what should be learned and thus failing exams and tests. Autonomy also decreased among students. In a situation characterized by stress, uncertainty, worries, loneliness, and several societal restrictions, they found it difficult to achieve a sense of acting autonomously. Ultimately, this study supports numerous others who have pointed out the importance of both teacher and peer support to students’ perceived competence, mental health, and well-being (e.g., Wentzel, 2009, 2017; Federici and Skaalvik, 2014; Ryan and Deci, 2017; Fitzgerald and Konrad, 2021; Suresh et al., 2021). Therefore, this study encourages the implementation of support strategies.

### Strengths and Limitations

A notable strength of this study is its empirical examination of associations that have not been previously tested. Building on a strong theoretical foundation with questionnaires in possession of good psychometric properties, this study expanded on previous studies by testing the associations between teacher and peer support, emotional state, and perceived competence among nursing students with SEM. This modeling technique accounts for random measurement errors, and the psychometric properties of the scales in the model were derived accurately. The SEM model included 19 items, requiring a sample of *n* ≥ 200. The listwise sample included 305 cases, representing a large sample (Brown, 2006; Hair et al., 2010). Missing values were infrequent.

Nevertheless, the findings of this study must be discussed, with some limitations in mind. First, the information input to the estimated SEM increases with more indicators per latent variable and more sample observations (Westland, 2010). Applying the three-indicator rule (Hair et al., 2010), the latent variables in the model were measured with only three and four indicators. However, the factor loadings were strong, supporting reliability. Despite a good fit, some alternative models might fit the data better or be more accurate. Regardless, the fit indices and composite reliability underpin the present results. We encountered no problems with discriminant and convergent validity. We found good factor loadings, indicating that the theoretical plausibility was good. All paths correspond well to the theoretical basis, which supports the findings. Second, the present sample included fewer men than women, reflecting the gender composition of the nursing student population in Norway. Moreover, first-year students were double as many as students in the second and third study years; plausibly, first-year students may experience a stronger need for teacher support than second- and third-year students. Third, the cross-sectional design did not allow us to determine causality. A longitudinal design would have allowed for changes to be assessed and compared over time. Fourth, the use of self-reported data carries a certain risk of the findings being based on common-method variance (Podsakoff et al., 2003).

## Conclusion

Nursing students must deal with high demands for theoretical learning and clinical competence development. The COVID-19 pandemic has utterly increased stress, worries, and uncertainty. This study found that teacher and peer support are remarkably significant to nursing students’ emotional states and perceived competence. Correspondingly, we conclude that teacher and peer support represent principal pedagogic resources that enhance both mental health and competence development among nursing students; compatibly, this manuscript suggests several pedagogical strategies. Furthermore, we encourage teachers to be attentive in identifying strategies to enhance teacher and peer support in nursing education, particularly in the context of a pandemic.

## Data Availability Statement

The original contributions presented in the study are included in the article/supplementary material, further inquiries can be directed to the corresponding author/s.

## Ethics Statement

Ethical review and approval was not required for this study because the ethical authorities in Norway state that when using NETTskjema (https://nettskjema.no/?lang=en) all ethical requirements are fulfilled. No ethical approval by an ethics committee was needed. No sensitive information is collected in this study. The patients/participants provided their written informed consent to participate in this study.

## Author Contributions

BU undertook the analyses and wrote the results section along with the introduction section. GH wrote the discussion section and the conclusion. TP and HT collected the data, gave feedback to the manuscript several times. All authors contributed to the drafts of the various sections of this article. All authors approved the submitted version.

## Conflict of Interest

The authors declare that the research was conducted in the absence of any commercial or financial relationships that could be construed as a potential conflict of interest.

## Publisher’s Note

All claims expressed in this article are solely those of the authors and do not necessarily represent those of their affiliated organizations, or those of the publisher, the editors and the reviewers. Any product that may be evaluated in this article, or claim that may be made by its manufacturer, is not guaranteed or endorsed by the publisher.

## Appendix

**APPENDIX TABLE 1:** The measurement items and their relevant values (mean, standard deviation, skewness, and kurtosis).

Concepts	Item	Mean	*SD*	Skewness	Kurtosis
Teacher relatedness	I get along with the teachers in the course.	4.18	1.46	–0.284	–0.382
	The teachers in the course care about me.	4.25	1.67	–0.350	–0.814
	I am close to the teachers in the course.	3.51	1.68	0.204	–0.993
Teacher guidance	The teachers help me so that i understand what i should focus on in the actual course.	4.41	1.60	0.383	–0.742
	When i work with different requirements (e.g., written submission, internship assignment, or bachelor thesis), the teachers direct me in how to perform the different tasks.	4.27	1.58	–0.263	–0.760
	When technical problems emerge, the teachers help me solve them.	3.70	1.56	–0.005	–0.506
Peer relatedness	I get along with the students in the course.	5.65	1.29	–1.510	2.554
	The students in the course care about me.	5.46	1.36	–1.152	1.233
	I am close to the students in the course.	5.28	1.61	–1.102	0.368
Peer guidance	The students help me so that i understand what i should focus on in the actual course.	5.69	1.36	–1.496	2.277
	When i work with different requirements (e.g., written submission, internship assignment, or bachelor thesis), the students direct me in how to perform the different tasks.	5.53	1.32	–1.194	1.482
	When technical problems emerge, the students help me solve them.	5.72	1.20	–1.439	3.031
Emotional state	During the pandemic……				
	I have felt more stressed than before.	4.77	1.70	–0.592	–0.577
	I have felt more anxious than before.	4.34	1.72	–0.292	–0.887
	I have felt lonelier than before.	4.47	1.89	–0.353	0.140
	I have felt sadder than before.	4.13	1.85	–0.157	1.173
Perceived competence	I feel confident in my ability to learn the material in this course.	4.50	1.43	–0.394	–0.566
	I am capable of learning the material in this course.	4.44	1.39	–0.362	–0.534
	I am able to achieve my goals in this course.	4.57	1.41	–0.580	–0.420
